# Cerebral and neural regulation of cardiovascular activity during mental stress

**DOI:** 10.1186/s12938-016-0255-1

**Published:** 2016-12-28

**Authors:** Xiaoni Wang, Binbin Liu, Lin Xie, Xiaolin Yu, Mengjun Li, Jianbao Zhang

**Affiliations:** 1 Key Laboratory of Biomedical Information Engineering of Education Ministry, Xi’an Jiaotong University, Xi’an, 710049 China; 2 Department of Information Engineering, Officers College of CAPF, Chengdu, 610213 China

**Keywords:** Mental stress, Cardiovascular response, Autonomic nerves system, Heart rate variability, Cortex

## Abstract

**Background:**

Mental arithmetic has been verified inducing cerebral and cardiovascular responses. However, the mechanism and sequential responses are still ambiguous. This study aims to reveal the mechanism of cardiovascular and autonomic responses and the related scalp positions that regulate the autonomic nerves system (ANS) during MA task.

**Methods:**

34 healthy male subjects aged between 19 and 27 years old (mean age 23.6 ± 2.3 years) were recruited in. Electrocardiogram, impedance cardiography, beat-to-beat blood pressure and electroencephalography were measured simultaneously and continuously during the experiments. And the analysis of time–frequency, approximate entropy and Pearson correlation coefficient were adopted. For statistical comparison, paired t test is utilized in the study.

**Results:**

The results showed that mental arithmetic task increased heart rate (from 72.35 ± 1.88 to 80.38 ± 2.34), blood pressure (systolic blood pressure: from 112.09 ± 3.23 to 126.79 ± 3.44; diastolic blood pressure: from 74.15 ± 1.93 to 81.20 ± 1.97), and cardiac output (from 8.71 ± 0.30 to 9.68 ± 0.35), and the mental arithmetic induced physiological responses could be divided into two stages, the first stage (10–110 s) and late stage (150–250 s). The high frequency power component (HF) of HRV decreased during MA, but the normalized low frequency power component (nLF) and LF/HF ratio of HRV increased only at the late stage. Moreover, during first stage, the correlations between approximate entropy of electroencephalography at Fp2, Fz, F4, F7 and the corresponding time–frequency results of HF were significant. During the late stage, the correlations between approximate entropy of electroencephalography at Fp2, Fz, C3, C4 and the corresponding nLF was significant.

**Conclusions:**

Our results demonstrated that (1) mental stress induces time-dependent ANS activity and cardiovascular response. (2) Parasympathetic activity is lower during mental arithmetic task, but sympathetic nerve is activated only during late stage of mental arithmetic task. (3) Brain influences the cardiac activity through prefrontal and temporal cortex with the activation of ANS during mental arithmetic.

## Background

Many evidences have indicated that mental stressors were associated with elevated incidences of coronary heart disease, stroke and hypertension [1, 2]. Besides, the roles of mental stress in arrhythmogenesis and sudden cardiac death are no longer confined to the realms of anecdote [3]. However, the mechanism linking stress and cardiovascular disease remains unclear, which arouses attention to the specific cardiovascular responses during mental stress.

Mental arithmetic (MA), which is most extensively used laboratory stressors to induce mental stress, can cause the changes of many physiological indices such as heart rate (HR), cardiac output (CO), and blood pressure (BP) [4, 5]. Studies based on heart rate variability (HRV) analysis declared that high frequency component (HF) decreases and ratio of LF-to-HF increases while the normalized low frequency component (nLF) remains controversial during the mental stress events [6–8]. In previous researches, the dynamic character of physiology mechanism was ignored since traditional methods were adopted. In fact, cardiovascular system is a non-stable and time-variant system and dynamic variation of cardiovascular during MA deserve to drawn more attention [9]. However, studies on the specific mechanism of time-variant cardiovascular response to MA is still unclear.

Mental arithmetic leads changes of the cortical potential, part of which are related to neural and cardiovascular response [10]. The neurocardiology, which focuses on anatomy and function connection between heart and brain, represents the intersection of neurology and cardiology [11, 12]. These brain–heart interactions help to explain the apparent randomness of sudden cardiac events and provide new insights into future novel therapies to prevent sudden death. Previous studies have given description about regulation of heart by ANS. This mechanism involves amygdala, insular cortex, parabrachial complex, hypothalamus, periaqueductal gray matter, nucleus of the tractus solitarius, and ventrolateral medulla [13–15]. In fact, the connection of heart and brain may be different within stresses. The toxic effects of mental stress on cardiovascular function remind us to focus on the specific mechanism about how cortex activity dynamically regulates ANS, which is still beyond present study.

This paper aims to discover the dynamic physiological response of cardiovascular system and specific cortex–cardiac connection with cardiac activity during MA task.

## Methods

### Subjects

34 healthy male subjects aged between 19 and 27 years old (mean age 23.6 ± 2.3 years) were recruited in. All individuals were asked to finish a preliminary questionnaire to provide detailed information on their medical history to ensure there is no cardiovascular related disease which may influence the function of ANS. None had any abnormal electrocardiography or abnormal arterial pressure. Subjects with medication that may affect brain or cardiovascular function were excluded.

The project was carried out with the approval of the Xi’an Jiaotong University Ethics Committee, and informed written consent was obtained from each subject after the experimental protocol had been explained.

### Experimental protocol

Our test was performed in a silent and temperature-controlled room (25 ± 1 °C) between 9 a.m. and 11 a.m. Smoking, caffeine, alcohol or heavy exercise should be abstained for 24 h before test.

Participants were seated comfortably facing a display monitor at about 50 cm far. They had a 10 min rest before the test to get a stable baseline. And then, subjects were instrumented for the task, which included 5 min of baseline and 5 min of MA. The experimental task was subtraction mode arithmetic. Volunteers were asked to continuously subtract 7 from a 3-digit number for 5 min and respond as quickly as they can. Subjects were requested to keep quiet and motionless during the test with their eyes open.

### Data acquisition

Electrocardiogram (ECG), impedance cardiography (ICG), beat-to-beat BP and electroencephalography (EEG) were measured simultaneously and continuously during the experiments. Surface Ag/AgCl electrodes were attached to the chest and limbs for ECG and ICG measuring. One-lead ECG and ICG was recorded by ECG100C and NICO100C (MP150, BIOPAC Systems, Inc., America) according to the electrode connections of the Acknowledge Software Guide for the measurements. Beat-to-beat arterial BP was logged continuously via a non-invasive finger photoplethysmography (FMS, Finapres Measurement Systems, Arnhem, Netherlands).

128-lead EEG was recorded by EGI system (EGI, Electrical Geodesics, Inc., America.). In the study, 18 electrodes were selected according to the international 10–20 lead systems. The electrode impedances were limited below 50 kΩ per site. All electrodes were referenced to linked ear lobe electrodes.

BP from the FMS analog output was send into the first input channel of MP150. Meanwhile, MP150 communicated with EGI through Net Amps 300 Clock Sync I/O. When recording of MP150 started, a trigger would send into EGI simultaneously. A marker was then located on EEG, which make it possible for synchronous acquisition of different instruments. All signals were sampled at 1000 Hz.

### Heart rate variability

Before R wave detection, the high frequency noise and the baseline drift of ECG were removed by wavelet transform. Wavelet modulus maximum algorithm was applied to detect the R–R intervals, the average accuracy of which was 99.85% [16]. The ECG signal, which had high and sharp P waves or ventricular arrhythmia may result in more detection errors than normal signals. To modify the false R-wave detection, an artifact modification and rejection was performed manually. R–R interval sequence was resampled to 2 Hz for further computation.

Heart rate variability (HRV) is extensively used as a non-invasive method in evaluating the autonomic regulation of sinus node. Compared with time domain parameters, frequency domain parameters give more detailed and accurate quantification of HRV. Two principal components were distinguished in the spectrum of HRV: low frequency (LF: 0.04–0.15 Hz), and high frequency (HF: 0.15–0.4 Hz) components [17]. HF component was considered to reflect parasympathetic activity while LF component turned out to be modulated by more complex neural and non-neural mechanisms [18]. The normalized LF(nLF), which defined as LF * 100/(LF + HF), is believed to reflect the sympathetic activity. The LF/HF ratio has been used to reflex the sympatho-vagal balance or the sympathetic modulations. Although the meaning of the parameters are controversial, [19], most researchers still choose to use these indexes before more accurate ones are found [20, 21].

The traditional spectral analysis methods includes fast Fourier transform (FFT) [22] and autoregressive algorithms [23]. But the spectral frequency components were integrals of power spectrum density over specific bands, which reflect the total effect of autonomic regulation over the whole period. But it’s of great importance to figure out the time variant HRV under specific experimental setting or to identify the onset of a certain disease, i.e. epilepsy [24]. Time–frequency methods offered the possibility to solve this problem. One of these methods named Morlet-wavelet transform (MWT) had been used in children with temporal lobe epilepsy, and had proved to be in line with classical parameters of HRV analysis [25].

To assess the nonlinear time-varying cardiovascular system, a time-variant and frequency-selective method named Morlet-wavelet transform (MWT) was applied in our study. Higher frequencies lead to a better time resolution and a worse frequency resolution. So the mother wavelet should be adapted to get a relatively better time as well as frequency resolution. In this study, mother wavelet ‘cmor3-1’ was adopted. Frequency-dependent complex analytic signal $y^{k} \left( {t,f_{n} } \right)$ of the HRV (*x* ^*k*^(*t*)) is computed by MWT [26], where k is the number of recordings we used in the calculation (K = 34). The time-variant power spectrum of every recording is $ps^{k} \left( {t,f_{n} } \right)$

1 $$ps^{k} \left( {t,f_{n} } \right) = \left| {y^{k} \left( {t,f_{n} } \right)} \right|^{2}$$

Then the average time-variant power spectrum PS of the 34 recordings can be estimated

2 $$PS(t,f_{n} ) = \frac{1}{K}\sum\limits_{k = 1}^{K} {ps^{k} (t,f_{n} )}$$

In order to compare with the traditional HRV spectrum analysis results, the band power (LF: 0.04–0.15 Hz, HF: 0.15–0.4 Hz) of the time-variant $PS\left( {t,f_{n} } \right)$ is defined as

3 $$LF(t) = \sum\limits_{{f_{n} = 0.04}}^{0.15} {PS(t,f_{n} )}$$

4 $$HF\left( t \right) = \sum\limits_{{f_{n = 0.04} }}^{0.15} {PS\left( {t,f_{n} } \right)}$$

As usual, LF is normalized by

5 $$nLF = \frac{LF}{LF + HF} * 100$$

Additionally the sympatho-vagal balance is

6 $${\text{PR}} = {\text{LF}}/{\text{HF}}$$

### Impedance cardiography analysis

Analysis of hemodynamic parameters, cardiac output (CO), stroke volume (SV) and systemic vascular resistance (SVR) were calculated by using noninvasive bioimpedance monitoring techniques, which based on the physiological signal ECG, BP and impedance signal.

Generally, impedance cardiology measurement is sensitive to motion artifacts and other noises. Disturbances in dz/dt (ICG) signal may cause the feature extraction to fail. So the wavelet based method was adopted to remove the noises of impedance signal before derivate (dz/dt) calculation. Sramek-estimates was adopted to obtain SV, CO and SVR [27] after the location of B point (opening of aortic valve) C point (maximum left ventricle flow) and X point (closing of aortic valve) on dz/dt.

### ApEn (approximate entropy)

Approximate entropy (ApEn) was first proposed by Pincus for measuring the complexity of the nonlinear system in 1991, and was demonstrated to be applicable to relatively short and noisy time-series data [28]. Till now, it has been used in a variety of biological data sets including endocrine hormone secretion data [29], epileptic EEG detection [30], heart rate [31] and respiration [32]. Pincus et al. [33] confirmed that ApEn could be stable when the data length is more than 1000 points. More detailed research pointed out that ApEn is sensitive to parameter choices especially when the data length N ≤ 200. As suggested, the length of our calculation window and the shift points were both 1000 points and m = 2, r = 0.2, which meant ApEn were get second by second. Preprocessing such as filtering and ocular artifact removing of EEG is completed by EEGlab toolbox of MATLAB before ApEn is done.

### Correlation of HRV and ApEn

Most research on stress induced cardiovascular responses have only involved HRV analyzing or EEG signal processing, but few of them focused on the interaction between two signals by Pearson correlation [10]. The results were ambiguous. In this study, an improved method was adopted. Data of ApEn of EEG and HRV were averaged of all subjects in baseline and MA respectively, so that individual differences and noise were removed while shared characteristics were amplified.

As described above, second-by-second ApEn of EEG and 2 Hz time-variant HRV had been calculated. Then average HRV parameters of each second were obtained for computation of Pearson correlation of HRV and ApEn.

### Statistical analysis

The difference between baseline and MA were compared with paired t test(SPSS software). Correlation coefficient was calculated in MATLAB. Statistical significance were represented as *(P < 0.05) and **(P < 0.01). All results are two-tailed. All data were represented as mean ± SEM.

## Results

### Cardiovascular and hemodynamic response during MA

Compared with baseline, MA significantly increased HR (HR: from 72.35 ± 1.88 to 80.38 ± 2.34 beats/min), CO (CO: from 8.71 ± 0.30 to 9.68 ± 0.35 L/min), systolic blood pressure (SBP: from 127.09 ± 3.23 to 141.79 ± 3.44 mmHg) and diastolic blood pressure (DBP: from 69.15 ± 1.93 to 76.20 ± 1.97 mmHg) while decreasing SVR (SVR: from 791.85 ± 32.88 to 801.64 ± 32.94 dyne s/cm^5^) (Table 1).

**Table 1:** Cardiovascular and hemodynamic indexes during MA

	Baseline	MA
HR (beat/min)	72.35 ± 1.88	80.38 ± 2.34**
SV (mL/beat)	121.13 ± 3.83	121.57 ± 4.36
CO (L/min)	8.71 ± 0.30	9.68 ± 0.35**
SBP (mmHg)	112.09 ± 3.23	126.79 ± 3.44**
DBP (mmHg)	74.15 ± 1.93	81.20 ± 1.97**
SVR (dyne s/cm^5^)	791.85 ± 32.88	801.64 ± 32.94

Values are given as mean ± SEM

Statistical significances are expressed by ** P < 0.01

**Fig. 1:**
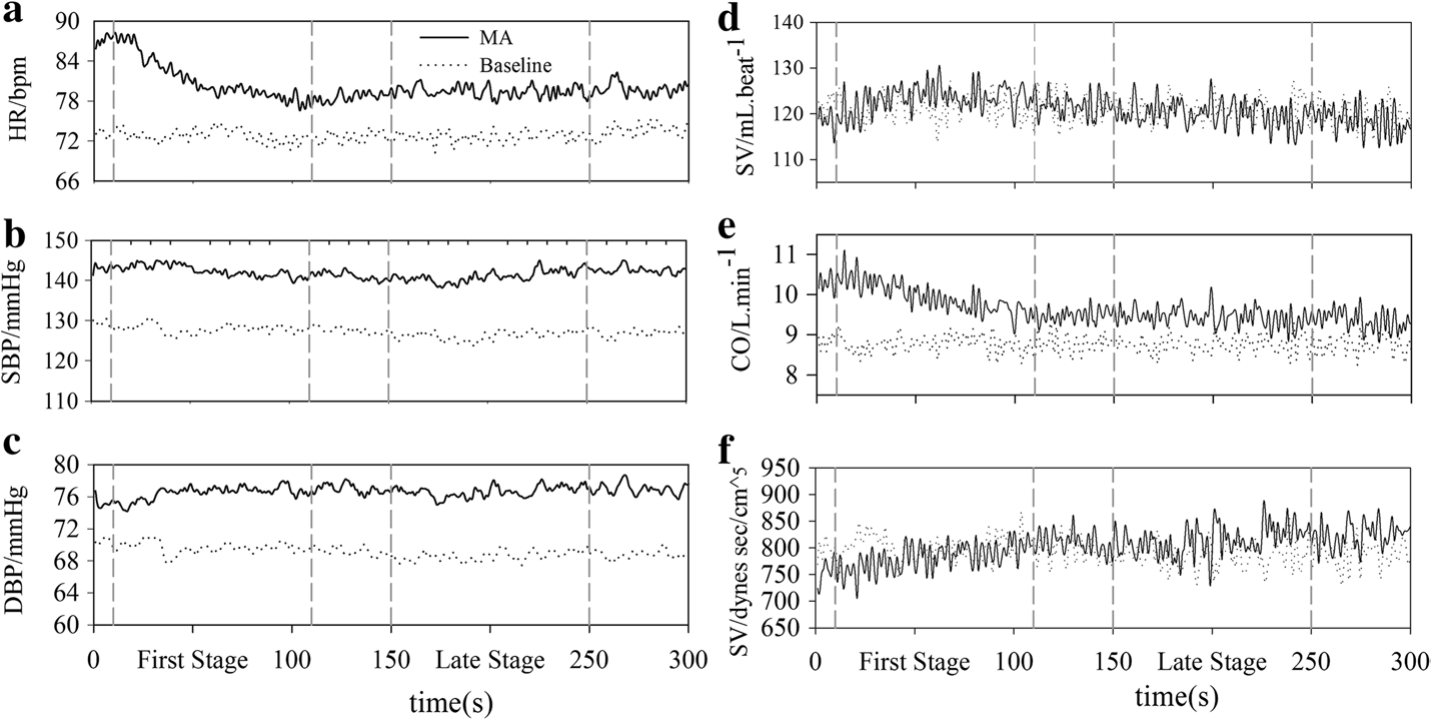
The time-variant cardiovascular and hemodynamic indexes. The baseline period is depicted with *dotted line* and MA is depicted with *solid line*

The results revealed that heart beating was quickened and BP was increased during MA. The tachycardia was combined with a significant rise of CO. Figure 1 showed the average dynamic process of above cardiovascular and hemodynamic parameters of 34 subjects. HR, CO, SBP and DBP raised rapidly at the beginning of MA task and then decreased to a stable level after 150 s, which was still much higher than the baseline.

### HRV time-variant spectral analysis

**Fig. 2:**
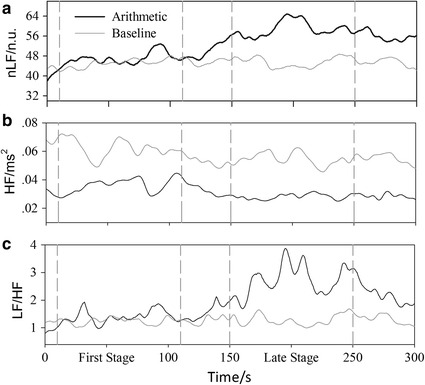
Grand-mean results (n = 34) of time–frequency related PS of RRI (MWT based). **a** The normalized LF, **b** HF, **c** the sympathetic-vagal balance(LF/HF). The nLF is shown by percentage. The baseline is depicted with *dotted line* and the MA is depicted with *solid line*

The time-variant spectral analysis of HRV was shown in Fig. 2, which depicted the change of the nLF, HF and LF/HF ratio during the 5 min baseline and MA test. HF remained relatively stable and significantly lower than the one of baseline group during the whole MA test. Compared with baseline, nLF and LF/HF ratio of MA group during 0–110 s did not significantly change, and then slowly increased. They finally got a stable level after 150 s. Based on our results of time-variant spectral analysis, there exist two stages for HRV of MA test, the first stage is 10–110 s and the late stage is 150–250 s. During first stage, HF (HF: from 0.06 ± 0.01 to 0.04 ± 0.005) significantly decreased while nLF and LF/HF ratio remained stable compared with baseline. For the late stage, nLF (nLF: from 45.13 ± 2.89 to 58.46 ± 3.30) and LF/HF ratio (LF/HF: from 1.27 ± 0.14 to 2.59 ± 0.43) dramatically increased while HF (HF: from 0.06 ± 0.01 to 0.03 ± 0.003) still decreased (Table 2).

**Table 2:** The statistical results of HRV

	Baseline	MA	
		First stage	Late stage
nLF (n.u.)	45.13 ± 2.89	46.43 ± 3.10	58.46 ± 3.30**
HF (ms^2^)	0.06 ± 0.01	0.04 ± 0.005*	0.03 ± 0.003*
LF/HF	1.27 ± 0.14	1.36 ± 0.21	2.59 ± 0.43**

Values are given as mean ± SEM

Statistical significances are expressed by * P < 0.05, ** P < 0.01

### Changes in scalp potential during MA

**Fig. 3:**
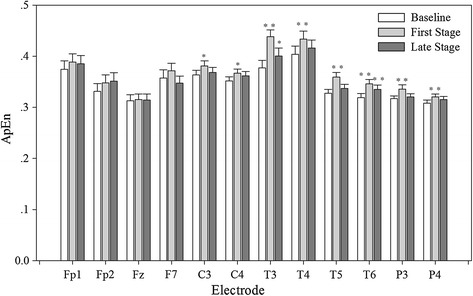
The ApEn change of scalp potential during baseline. Statistical significances (n = 34) are expressed by *P < 0.05, **P < 0.01

Mental arithmetic would induce cerebral activity. Figure 3 showed ApEn of EEG during first and late stages of MA task. ApEn at C3, C4, T3, T4, T5, T6, P3 and P4 electrodes significantly increased during first stage for MA task. For the late stage, MA made ApEn at T3 and T6 significantly increase.

### Relationship between scalp potential and autonomic activity

The cerebral activity could induce autonomic response and the relative relationship between HRV and ApEn was analyzed (Table 3). As the correlation results of LF/HF ratio component and ApEn were similar to nLF, the results only involved the correlation coefficients of nLF and HF with ApEn. There seemed no correlation between ApEn and HRV for the baseline except F7 position. During MA task, the correlation coefficients between ApEn and nLF were still not significant at the first stage except Fp1position. However, significant positive correlation were detected at Fp1, Fp2, Fz, F7, C3 and C4 electrodes during the late stage of MA task. Considering of the relationship between HF and ApEn of EEG, positive relationships were discovered at Fp1, Fp2, Fz, F4 and F7 during first stage while negative relationships were found at Fp1, Fz, F7 and C4 during late stage.

**Table 3:** The correlation coefficients between ApEn and HRV

	nLF_ApEn			HF_ApEn		
	Baseline	First stage	Late stage	Baseline	First stage	Late stage
Fp1	0.14	−0.20*	0.26**	0.02	0.42**	−0.25**
Fp2	0.07	−0.16	0.28**	0.01	0.31**	−0.16
Fz	0.10	−0.11	0.22*	0.13	0.34**	−0.38*
F3	0.10	−0.07	−0.09	0.10	0.11	−0.10
F4	0.13	0.04	0.12	0.04	0.44**	−0.08
F7	0.25**	−0.05	0.21*	0.09	0.30*	−0.26*
F8	0.14	0.01	0.10	0.13	0.12	−0.12
C3	0.13	0.05	−0.25*	0.03	−0.13	0.11
C4	0.01	−0.02	0.21*	−0.00	0.04	−0.41**
T3	0.08	0.03	−0.13	−0.14	0.12	−0.02
T4	0.07	−0.09	−0.02	−0.03	0.17	0.02
T5	0.01	0.04	−0.10	−0.08	0.03	−0.11
T6	0.06	0.06	0.10	0.05	−0.03	−0.18
P3	0.07	0	−0.16	0.01	0.01	−0.03
P4	−0.03	0.02	−0.02	0.07	−0.10	−0.17
Pz	0.09	0.12	0	0.13	0.15	−0.14
O1	−0.11	−0.12	0.02	−0.01	0.10	−0.07
O2	0.01	−0.10	0.19	0.03	0.09	−0.30

Values are given as mean ± (n = 34)

Statistical significances are expressed by * P < 0.05, ** P < 0.01

## Discussion

In order to extract time-varying information of detect the specific mechanism of dynamic cardiovascular response to MA, a comprehensive examination involving measurements of ECG, ICG, BP, EEG and the analysis of time–frequency, approximate entropy and Pearson correlation coefficient were adopted. The study revealed three important and novel findings. First, mental stress induces time-dependent cardiovascular response. Second, parasympathetic activity is lower during MA task, but sympathetic nerve is activated only during late stage of MA task. Third, brain induces the cardiac activity through prefrontal and temporal cortex with the activation of ANS during MA.

The discussion below is based on average group study results of all 34 subjects, which is adopted by a large number of studies so that common characteristics can be amplified while individual differences can be counteracted. The conclusions summarized below are suitable for most of individual.

### Cardiovascular responses during MA

MA increases HR, BP, and CO [34]. Present study not only obtained similar results (Table 1) but also extracted time-varying information of cardiac response (Fig. 1). Consistent with our hypotheses, the study strengthened the rationale and dynamic performance. Some studies argued that the increases of HR and BP during mental stress were a response mediated by the release of norepinephrine, which is related to sympathetic activation [35–37]. However, time-varying analysis of HRV in present study (Fig. 2) provide a new sight on understanding of ANS regulation process. Based on our dynamic results, MA altered cardiovascular indices immediately when test started and accompanied with parasympathetic withdrawal rather than sympathetic activation. The changes of cardiovascular system during MA are induced by two major pathways: parasympathetic withdrawal and cardiac self-regulation.

As we all know, parasympathetic activity dominates SA at rest and makes the heart maintain HR at 75 bpm, which is greatly slower than SA node’s intrinsic rate [38]. When MA started, parasympathetic nerve retreated immediately and then leaded to cardiovascular responses, not only the increase of BP and limbs blood flow, but also the combination of greatly increase of HR and the transient slight decrease of SV, which cause the increase of CO.

Besides, MA revved up the metabolism of the brain, combined with cerebral blood flow increased [6], and then enhanced venous return and venous pressure. According to the Starling’s Law of the heart, SV would increase with the gain of venous pressure, and cause the raise of BP [39].

### Autonomic reactivity during MA

ANS responses on MA are diverse according to existing results. Mental stress has been reported to increase [40, 41], decrease [42, 43], and does not change [44] muscle sympathetic nerve activity (MSNA) during MA. In the study, MA consistently triggers an increase in HR, BP and CO, however, the cardiac SNS is not activated consistently. Instead, the break of the balance of SNS only happened in the late stage of the experiment according to the time–frequency result of HRV. Our time-varying results showed that cardiovascular responses during MA are time-variant and there exists two different stages for MA-induced cardiovascular responses in 5 min task (Table 2; Fig. 2): first stage (10–110 s) and late stage (150–250 s).

At the beginning of MA (first stage), sympathetic nerves were not activated while the parasympathetic withdrawal happened. At this time, the increase of HR, BP and CO is generated by the parasympathetic withdrawal as well as self-regulation of the cardiovascular system as described above.

As MA continues (late stage), SNS was then activated and got another stable state. However, the augment of sympathetic nerves activity did not cause further changes of cardiovascular indices except for the slight increase of SVR. Recent study demonstrated that increased sympathetic activity, which were induced in healthy volunteers following a series of fatigue-inducing mental tasks, made no contribution to cardiovascular integrating [45]. We speculated that increase of SNA may be associated with fatigue of brain during this stage.

### Cerebral regulation during MA

Cerebral positions related to regulation of cardiovascular during MA were examined in this study. Previous study showed that MA induced significant increase of ApEn at P3, P4, Pz electrodes [10] and it was also found that the anterior cingulate and insular cortex were activated during a variety of tests [46], MA [47], emotion and attention [48]. Prefrontal, anterior, and midcingulate and insular cortices are implicated in sympathetic regulation [13]. In this study, correlation coefficients between ApEn and HRV components revealed the brain-cardiac connection in a way. For the baseline, the relationship of ApEn with nLF was only found at F7 and it indicates that left temporal cortex is possibly implicated in the connection of cortex with cardiac activity. Gray et al. [49] pointed out that the amplitude of the heartbeat-evoked potential in the left temporal region reflected the proarrhythmic status of the heart and the activated position is consistent with ours. Moreover, cerebral regulation of ANS in 5 min MA also can be divided into two different stages.

During the first stage, MA induced the increase of HF and the break of balance of ANS only happened in parasympathetic part, which lead to the cardiovascular response. ApEn of EEG was significantly related with HF at Fp2, F4 and Fz electrodes. The correlation analysis revealed that right prefrontal cortex was possibly involved in parasympathetic regulation. Wong et al. [50] pointed out that the ventral medial prefrontal cortex is involved in modulating the vagal efferent outflow to the heart and the suppression of its activity elevates cardiovascular arousal in conscious humans and their conclusion is strengthen our results.

For late stage, both sympathetic and parasympathetic regulation were influenced by cortex and sympathetic increase play a predominant role. The correlation coefficients of ApEn and nLF at electrode Fp2, C3 and C4 (prefrontal cortex and positions near insular cortex) are significant. Among them, the ApEn of prefrontal cortex and right insular cortex (C4) have positive correlation with the nLF component of HRV, while the left insular (C3) has negative correlation with nLF. In fact, in 1992, Stephen designed a stimulation experiment to insular cortex and found that stimulation of the left insular cortex, bradycardia and depressor responses were more frequently produced than tachycardia and pressor effects. The opposite result was also gotten at right insular cortex [15]. This report is concordant with our results. We speculate that there is right-sided dominance for sympathetic effects. Activation of cortex near C4 and inhibition of C4 are possibly involved in sympathetic activation.

The results revealed the specific areas that influence SNS activity and it demonstrates the lateralization of the responses for a cortical site.

## Conclusion

MA leads to the augment of HR, BP and CO which is caused by parasympathetic withdrawal and cardiac self-regulation. Prefrontal cortex was involved in PNA regulation. As MA continued, SNA increased in late stage maybe due to fatigue of brain, which didn’t result in further change of cardiovascular. Prefrontal cortex and right insular cortex activation have relationship with SNA increase. The converse result is discovered in left insular cortex.

To our knowledge, it’s the first time for verifying the dynamic process of cardiac activity induced by MA. Besides, our demonstrations will give a new sight in understanding the scalp sites in autonomic nerves activity regulation as well as a comprehension of the pathways from the cortex to ANS. Furthermore, our findings may lead to further studies of the new methods for the treatment of cardiovascular diseases.

## Abbreviations

ANS: autonomic nerves system
BP: blood pressure
CBF: cerebral blood flow
CO: cardiac output
HRV: heart rate variability
ICG: impedance cardiography
LVEF: left ventricular ejection fraction
LVET: left ventricular ejection time
MA: mental stress
PEP: pre-ejection period
PNA: parasympathetic nerves activity
PNS: parasympathetic nerves system
SNA: sympathetic nerves activity
SNS: sympathetic nerves system
SV: stroke volume
